# Ischemia reperfusion injury promotes recurrence of hepatocellular carcinoma in fatty liver via ALOX12-12HETE-GPR31 signaling axis

**DOI:** 10.1186/s13046-019-1480-9

**Published:** 2019-12-12

**Authors:** Faji Yang, Yuheng Zhang, Haozhen Ren, Jinglin Wang, Longcheng Shang, Yang Liu, Wei Zhu, Xiaolei Shi

**Affiliations:** 1 Department of Hepatobiliary Surgery, Affiliated Drum Tower Hospital of Nanjing University Medical School, 321, Zhongshan Road, 210008 Nanjing, Jiangsu Province, China; 2 Department of Anesthesiology, Affiliated Drum Tower Hospital of Nanjing University Medical School, 321, Zhongshan Road, 210008 Nanjing, Jiangsu Province, China

**Keywords:** Ischemia reperfusion injury, ALOX12, 12-HETE, HCC recurrence, GRP31

## Abstract

**Background:**

Ischemia reperfusion injury (IRI) has been shown to increase the risk of tumor recurrence after liver surgery. Also, nonalcoholic fatty liver disease (NAFLD) is associated with increased HCC recurrence. ALOX12–12-HETE pathway is activated both in liver IRI and NASH. Also, ALOX12–12-HETE has been shown to mediate tumorigenesis and progression. Therefore, our study aims to investigate whether the ALOX12–12-HETE-GPR31 pathway involved in IRI induced HCC recurrence in NAFLD.

**Methods:**

HCC mouse model was used to mimic the HCC recurrence in NAFLD. Western Blot, qPCR, Elisa and Immunofluorescence analysis were conducted to evaluate the changes of multiple signaling pathways during HCC recurrence, including ALOX12–12-HETE axis, EMT, MMPs and PI3K/AKT/NF-κB signaling pathway. We also measured the expression and functional changes of GPR31 by siRNA.

**Results:**

ALOX12–12-HETE pathway was activated in liver IRI and its activation was further enhanced in NAFLD, which induced more severe HCC recurrence in fatty livers than normal livers. Inhibition of ALOX12–12-HETE by ML355 reduced the HCC recurrence in fatty livers. In vitro studies showed that 12-HETE increased the expression of GPR31 and induced epithelial-mesenchymal transition (EMT) and matrix metalloprotein (MMPs) by activating PI3K/AKT/NF-κB pathway. Furthermore, knockdown of GPR31 in cancer cells inhibited the HCC recurrence in NAFLD.

**Conclusions:**

ALOX12–12-HETE-GPR31 played an important role in HCC recurrence and might be a potential therapeutic target to reduce HCC recurrence after surgery in fatty livers.

## Background

Hepatocellular carcinoma (HCC) is the fifth most common cancer and the third leading cause of cancer related death worldwide [1]. Although chronic hepatitis B virus (HBV) and HCV infection account for most cases of HCC, nonalcoholic fatty liver disease (NAFLD) has been recently reported as an increasing etiology for HCC [2]. It is estimated that NAFLD-related HCC accounts for more than 13% of HCC cases in US [3]. Surgical resection and liver transplantation are the common treatment for HCC. Despite the advances in surgical techniques and surgical care, the survival rates for HCC patients have been unsatisfactory due to the high recurrence rates. About 40–70% of the patients suffer from postoperative recurrence within 5 years [4], which reduces the patient survival [5]. Recent studies found that even with successful surgical removal, NAFLD is associated with an increased recurrence of HCC [6, 7]. Ischemia and reperfusion injury (IRI) is also considered to increase the risk of tumor recurrence after liver transplantation or surgical resection [8–10]. Moreover, our previous study indicated that PARP-1 promotes tumor recurrence after warm ischemic liver graft transplantation via neutrophil recruitment and polarization [11]. However, the mechanism of tumor recurrence in NAFLD is still unclear.

Arachidonate-12-Lipoxygenase (ALOX12) is highly expressed in platelets and is extensively expressed in various cell types, including hepatocytes [12]. Arachidonic acid (AA), a polyunsaturated omega-6 fatty acid, is a component of the phospholipid domain for most cell membranes [13]. AA is cleaved from membrane phospholipids by cytosolic phospholipase A2 and metabolized to hydroxyeicosatetraenoic acids (HETEs) through the lipoxygenase pathway. A recent study found that ALOX12–12-HETE was activated during liver IRI and the block of 12-HETE production inhibited IRI-induced liver dysfunction, inflammation and cell death [14]. In addition, serum metabolomic analysis revealed that 16:0-, 18:0-, and 18:1-LPC were significantly decreased and 12-HETE was markedly increased in mice with fatty liver disease [15]. However, whether ALOX12–12-HETE was activated during IRI in fatty livers remains unknown. Furthermore, ALOX12 can convert AA to 12-HETE, which plays an important role in cancer [16]. ALOX12–12-HETE has been reported to promote prostate carcinoma progression [17]. It has also been shown to mediate the invasion of intrametastatic lymphatic vessels and propagate lymph node metastasis in a mouse model of mammary carcinoma [18]. However, the role of ALOX12–12-HETE in HCC as well as tumor recurrence has not been investigated.

Epithelial-mesenchymal transition (EMT) is a key step for tumor invasion and metastasis [19]. Tumor cells develop invasive and metastatic abilities during the EMT process, and migrate to different sites via the circulatory system [19]. The EMT program has also been implicated in HCC recurrence [20]. Matrix metalloproteins (MMPs) play key roles in a variety of biological processes, including matrix degradation, growth factor receptor signaling, angiogenesis, cell adhesion and apoptosis [21]. MMPs have been recognized as major contributors to the proteolytic degradation of extracellular matrix during tumor invasion [22], and they can also contribute to tumor recurrence [23, 24]. However, the role of MMPs and EMT in HCC recurrence of fatty livers has not been well studied.

In the present study, we found that ALOX12–12-HETE was activated during liver IRI and its activation was enhanced in fatty liver, which induced more severe tumor recurrence in fatty liver than normal liver. Moreover, the GPR31 dependent EMT and MMPs induced by 12-HETE played an important role in the invasion ability of circulation tumor cells. Our study is the first to investigate the roles of ALOX12–12-HETE-GPR31 in HCC recurrence and provide a promising strategy to reduce the HCC recurrence in liver surgery.

## Methods

### Cell culture

The human HCC cell line (bel-7402 and Huh7), mouse HCC cell line (Hepa1–6) and mouse normal cell line (AML12) were purchased from Cell Bank of Type Culture Collection of the Chinese Academy of Sciences (Shanghai Institute of Cell Biology). Huh7 and Hepa1–6 were maintained in Dulbecco’s modied Eagle’s medium (DMEM) supplemented with 10% FBS (Gibco, USA) at 37 °C and 5% CO2, and the bel-7402 cell was cultured in RPMI-1640 medium (Gibco, USA). AML12 cell was maintained in DMEM/Ham’s F-12 Nutrient Mixture supplemented with 10% FBS (Gibco, USA) at 37 °C and 5% CO2. Cell lines were authenticated by short tandem repeats (STR) profiling and confirmed to be mycoplasma negative.

To evaluate the hypoxia-reoxygenation (H/R) injury in vitro, AML12 cells were treated with palmitic acid (PA, Sigma-Aldrich, USA) for 24 h followed by fluxed with 95% N_2_/5% CO_2_ without FBS and maintained at 37 °C for 16 h. For reoxygenation, cells were transferred to a 95% air/5% CO_2_ gas mixture with 10% FBS for 2 h.

### Animals

Experiments were conducted on male C57BL/6 J mice, which were purchased from the Animal Center of the Affiliated Drum Tower Hospital of Nanjing University Medical School and housed under specific pathogen-free conditions. The animal experiments were approved by the Institutional Animal Care and Use Committee of Nanjing University, China, based on the NIH Guide for the Care and Use of Laboratory Animals. All efforts were made to minimize suffering.

Male C57BL/6 mice (3–4 weeks old) were fed with high fat diet (D12492; Research Diets, New Brunswick, USA) for 14 weeks to induce steatosis.

### Hepatic IRI and hepatocellular carcinoma mouse model

Hepatocellular carcinoma model was established as previously described [7]. In brief, Hepa1–6 cells were detached by trypsinization and incubated for 1 h in a non-adherent Petri dish. Then the cells were prepared in a suspension containing 5 × 10^5^ dells per 200 μl PBS. The mouse 70% hepatic IRI mouse model was established as previously described [25] and the Hepa1–6 cells suspension was gently injected into the spleen with a 29-G needle as soon as the arterial clip was removed. Successful inoculation was confirmed by the absence of bleeding, and by visualization of splenic blush, liquid flow in the splenic vein, and transient liver discoloration. Splenectomy was performed after allowing the cells to circulate for 15 min. Three weeks later, the mice were sacrificed. The liver samples were harvested and preserved in 4% formalin or snap frozen in liquid nitrogen.

### Western blot analysis

Proteins were electrophoresed by SDS/PAGE (12% or 10% gel) and the blots were incubated overnight with primary antibody. The following primary antibodies were used: anti-ALOX12 (Santa-Cruz, sc-365,194), anti-E-Cadherin (Cell signal Technology, #14472), anti-N-Cadherin (Cell signal Technology, #13116), anti-Vimentin (Cell signal Technology, #5741), anti-Snail (Cell signal Technology, #3879), anti-Slug (Cell signal Technology, #9585), anti-MMP2 (Abcam, ab37150), anti-MMP7 (Abcam, ab5706), anti-MMP9 (Abcam, ab38898), anti-MMP13 (Abcam, ab39012), anti-GPR31 (Abcam, ab75579), anti-PI3K (Cell signal Technology, #4249), anti-AKT (Abcam, ab179463), anti-AKT (phospho T308, Abcam, ab38449), anti-NF-κB (Abcam, ab16502), anti-NF-κB (phospho S536, Abcam, ab86299), anti-GAPDH (Abcam, ab181602).

### Enzyme-linked immunosorbent assay (ELISA)

The levels of 12-HETE in mouse serum and cell culture supernatants were measured using commercially available ELISA kits (Abcam, USA) according to the manufacturer’s instructions.

### GPR31 knockdown

The siRNA sequences of GPR31 are as follows: TCCACACCCTGACCCGGAAC (human) and CCGTCTCAAAGTATGGAAGCCTTAT (mouse). Cells were transiently transfected with siRNA using Lipofectamine2000 (Invitrogen, USA) according to the manufacturer’s instructions.

### Quantitative real-time polymerase chain reaction (qRT-PCR)

RNA was extracted from liver samples and cells with TRIzol™ reagent (Life Technologies, USA) according to the manufacturer’s instructions. Reverse transcription was performed with PrimeScript™ RT Master Mix (Takara, Japan) according to the manufacturer’s instructions. qRT-PCR was performed using TB Green™ Premix Ex Taq™ (Takara, Japan) and ABI PRISM 7500 real-time PCR System (Applied Biosystems, USA). Primers used for qPCR are shown in Additional file 1: Table S1.

### Hematoxylin-eosin (HE) staining

Paraffin liver sections (5 μm) were stained with hematoxylin and eosin (HE) for histological evaluation of HCC recurrence based on standard pathology methods.

### Immunocytofluorescence (ICF)

Cells and frozen liver sections (4 μm) fixed with acetone were penetrated with 0.3% Triton for 15 min. Then the sections were block with 10% fetal sheep serum, followed by incubation with primary antibodies overnight at 4 °C. After washing, sections or cells were incubated with corresponding secondary antibodies, followed by incubation with DAPI. After mounting, the slides were observed under immunofluorescence microscope. The following antibodies were used: anti-ALOX12 (Santa-Cruz, sc-365,194), anti-Vimentin (Cell signal Technology, #5741), anti-MMP9 (Abcam, ab38898), Goat Anti-Mouse IgG H&L (Alexa Fluor® 488) (Abcam, ab150117).

### Statistical analysis

Statistical analysis was performed using GraphPad Prism software version 6.0. All data are expressed as mean ± standard error of the mean (SEM). Normally distributed data were tested by Student’s t-test. *P*-value less than 0.05 was considered statistically significant.

## Results

### IRI induced more severe HCC recurrence in fatty liver

**Fig. 1:**
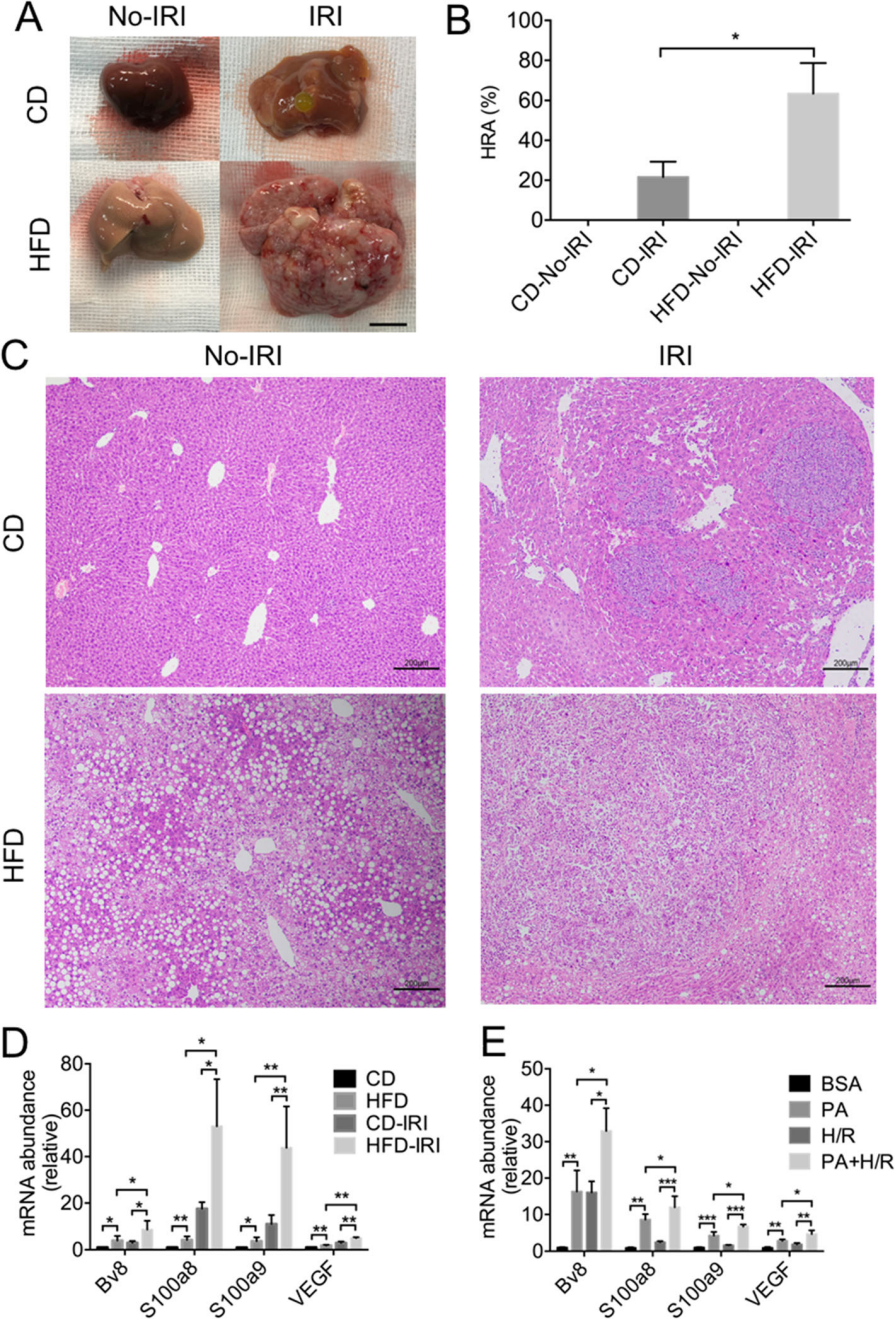
IRI induced more severe HCC recurrence in fatty liver. **a, b** Representative images and hepatic replacement area (HRA) of HCC recurrence in CD and HFD mice inoculated with Hepa1–6 cells with or without IRI. Scale bars, 1 cm. (*n* = 3–4 per group) **c** Representative H&E staining of liver sections in CD and HFD mice inoculated with Hepa1–6 cells with or without IRI. Scale bars, 200 μm. **d** qPCR analysis of *Bv8, S100a8, S100a9* and *VEGF* in CD and HFD feeding mice after IRI. (*n* = 4–5 per group) **e** qPCR analysis of *Bv8, S100a8, S100a9* and *VEGF* in AML12 cells stimulated with PA (50 μM) followed by HR. (n = 4–5 per group) Data are mean ± SEM, *p < 0.05, **p < 0.01, ***p < 0.001 by unpaired Student’s t- test

To characterize the effect of liver steatosis on the implantation of circulating HCC cells, mice fed with normal diet (control diet, CD) and high fat diet (HFD) were splenic inoculated with 5 × 10^5^ Hepa1–6 cells after 60 min of ischemia. For both CD and HFD groups, the livers without IRI exhibited almost no HCC recurrence while the livers with IRI exhibited different degrees of tumors (Fig. 1a and b). Moreover, the control livers only exhibited sparse single tumor nodules, whereas the fatty livers showed numerous and confluent tumor nodules, suggesting that liver steatosis promoted the implantation of circulating HCC cells (Fig. 1a and b). This result was also confirmed by HE staining on the liver sections (Fig. 1c). Moreover, the expression of the pre-metastatic milieu related factors *Bv8, S100a8, S100a9* and *VEGF* were all upregulated during IRI, and were further increased in fatty livers (Fig. 1d). We also simulated the fatty liver environment with IRI in vitro by stimulating AML12 cells with palmitic acid (PA) for 24 h followed by hypoxia/re-oxygenation (H/R). All the pre-metastatic milieu related factors were upregulated in AML12 cells, which agreed with the in vivo experiments (Fig. 1e). Overall, compared with the control group, the mice fed with HFD exhibited significantly higher HCC recurrence after IRI.

### ALOX12–12-HETE was activated during liver IRI in NAFLD

**Fig. 2:**
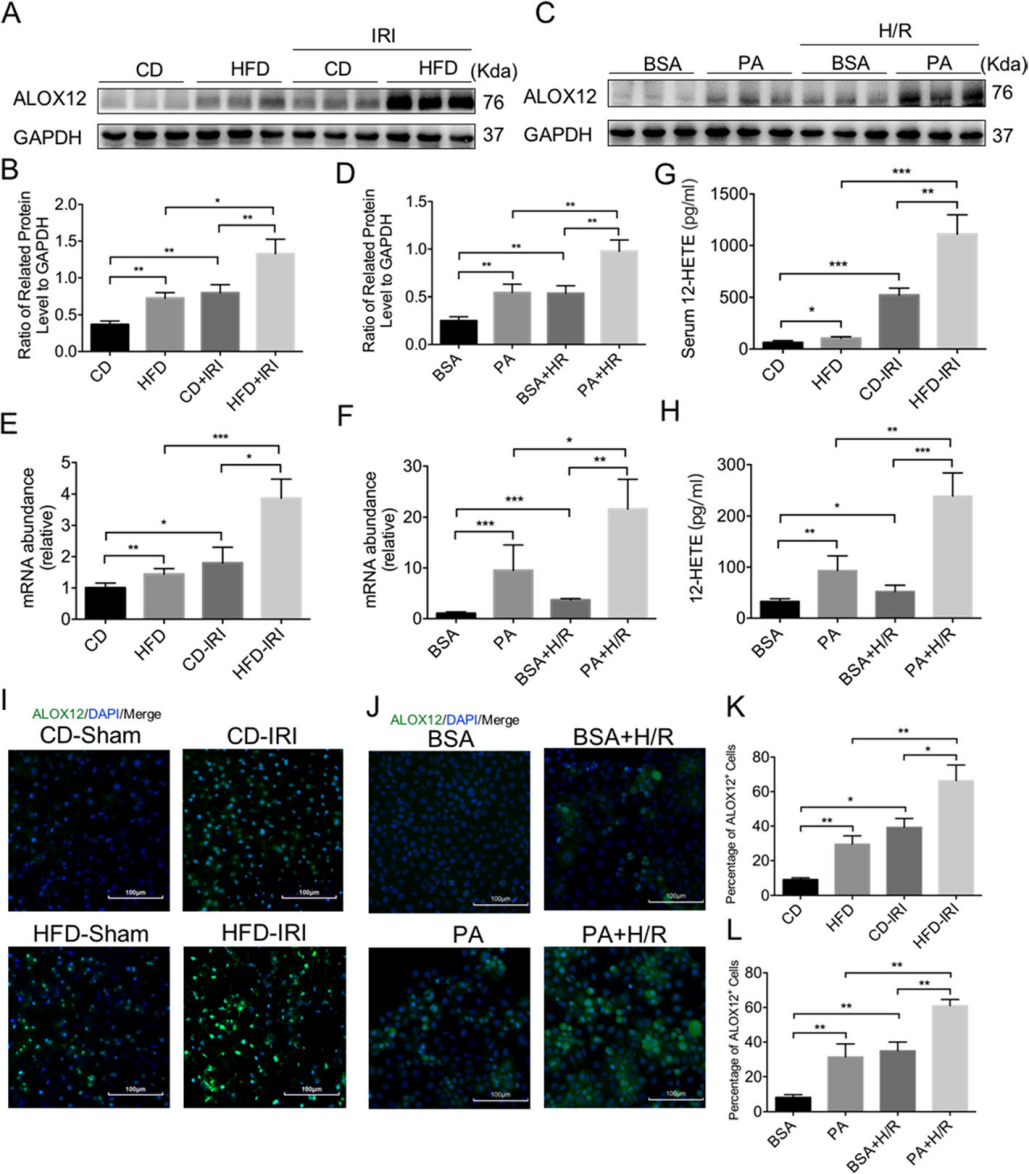
ALOX12–12-HETE was activated during liver IRI in NAFLD. **a, b** Immunoblot analysis of ALOX12 in CD and HFD feeding mice after IRI. Protein levels were normalized to GAPDH and analyzed. (*n* = 3 per group) **c, d** Immunoblot analysis of ALOX12 in AML12 cells stimulation with PA followed by HR. Protein levels were normalized to GAPDH and analyzed. (n = 3 per group) **e.** qPCR analysis of *ALOX12* in CD and HFD feeding mice after IRI. (n = 4–5 per group) **f.** qPCR analysis of *ALOX12* in AML12 cells stimulation with PA followed by HR. (n = 4–5 per group) **g, h** 12-HETE in serum and in cell supernatant were measured. (n = 4–5 per group) **i-l** Representative immunofluorescence staining of ALOX12. Scale bars, 100 μm. (n = 3 per group) Data are mean ± SEM, *p < 0.05, **p < 0.01, ***p < 0.001 by unpaired Student’s t- test

Recent studies have shown that ALOX12–12-HETE pathway was activated both in liver IRI and NASH, but whether ALOX12–12-HETE pathway was activated during IRI in NAFLD remains unknown. We found that the protein and mRNA levels of ALOX12 were upregulated during IRI in both fatty livers and normal livers, but the upregulation was more significant in fatty livers (Fig. 2a, b and e). Consistently, ALOX12 was also activated in the in vitro experiment (Fig. 2c, d and f). Moreover, 12-HETE, the metabolite of ALOX12, was also increased more obviously in fatty livers than normal livers during IRI, both in vivo and in vitro (Fig. 2g and h). We also measured ALOX12 of hepatocytes by immunofluorescence (Fig. 2i-l), and found that the ALOX12 staining was increased in fatty livers with IRI. Therefore, the ALOX12–12-HETE pathway may play a role in IRI induced HCC recurrence in NAFLD.

### ML355 reduced HCC recurrence by inhibiting ALOX12–12-HETE pathway

**Fig. 3:**
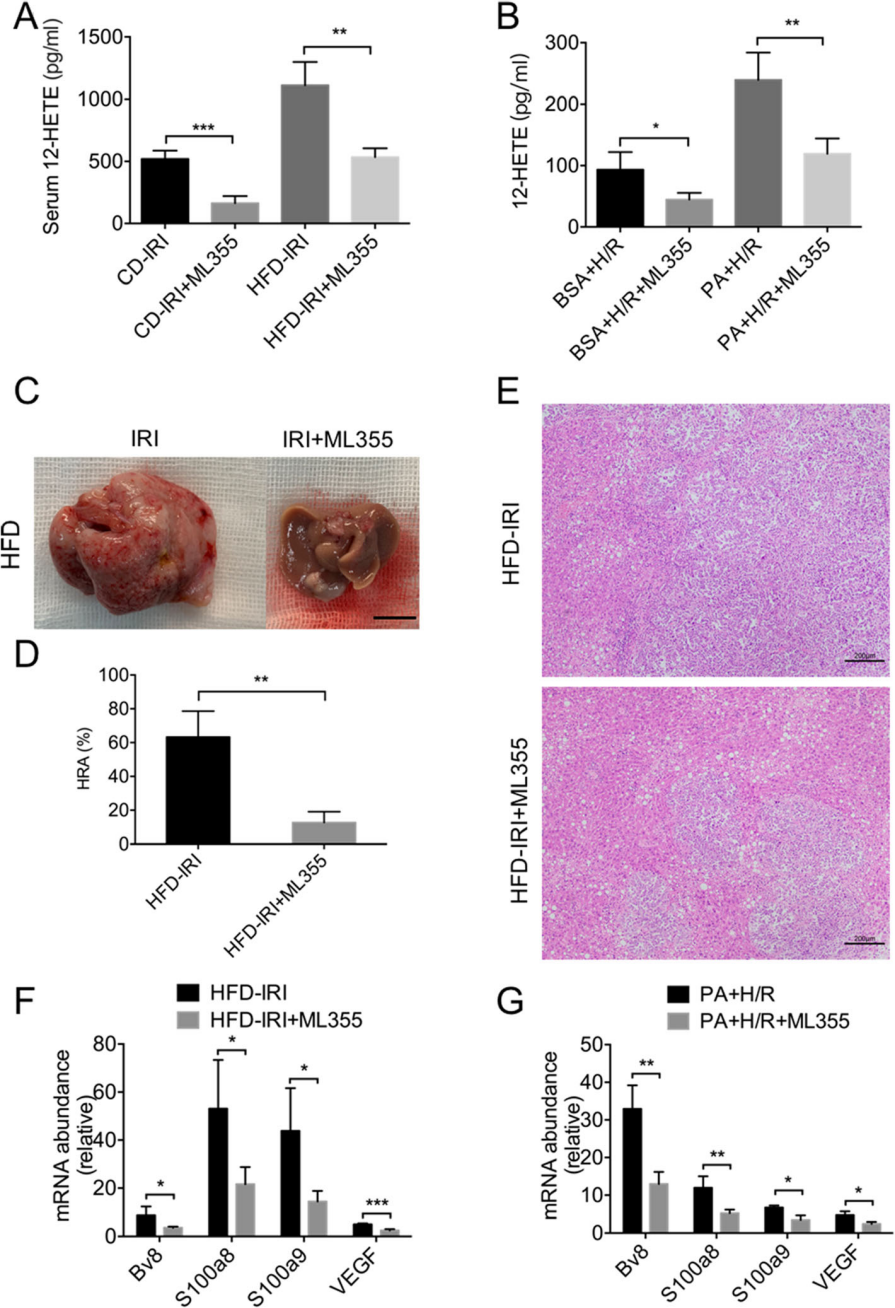
ML355 reduced HCC recurrence by inhibiting ALOX12–12-HETE pathway. **a, b** 12-HETE in serum and in cell supernatant were measured. (*n* = 4–5 per group) **c, d.** Mice was pretreated with ML355 (3 mg/kg body weight, MCE, USA) at 1 h before ischemia. Representative images and HRA of HCC recurrence in HFD mice pretreated with ML355 or PBS followed by IRI and inoculated with Hepa1–6 cells. Scale bars, 1 cm. (*n* = 3–4 per group) **e.** Representative H&E staining of liver sections of HCC recurrence in HFD mice pretreated with ML355 or PBS followed by IRI and inoculated with Hepa1–6 cells. Scale bars, 200 μm. **f.** qPCR analysis of *Bv8, S100a8, S100a9* and *VEGF* in HFD mice pretreated with ML355 or PBS followed by IRI. (n = 4–5 per group) **g.** qPCR analysis of *Bv8, S100a8, S100a9* and *VEGF* in AML12 cells pretreated with ML355 or PBS and stimulated with PA followed by HR. (n = 4–5 per group). Data are mean ± SEM, *p < 0.05, **p < 0.01, ***p < 0.001 by unpaired Student’s t- test

As the above results showed, ALOX12–12-HETE pathway was activated by IRI and the activation was further enhanced in NAFLD. Recent studies have shown that this pathway can promote tumor progression in various cancers. Therefore, we next asked whether ALOX12–12-HETE pathway promoted HCC recurrence in fatty livers. Mice were treated with ML355, an ALOX12 inhibitor, before IRI, and their serum 12-HETE levels were decreased after IRI (Fig. 3a). Also, ML355 reduced 12-HETE level in the supernatant of AML12 cells stimulated by PA and H/R (Fig. 3b). We also found that the HCC recurrence in NAFLD was significantly inhibited by ML355 (Fig. 3c). The fatty livers exhibited decreased number and volume of nodules, as verified by HE staining (Fig. 3d and e). Moreover, the pre-metastatic milieu related factors, *Bv8, S100a8, S100a9* and *VEGF* were all significantly reduced in fatty liver after ML355 treatment (Fig. 3f). This change was also consistent with the in vitro experiment (Fig. 3g). Collectively, these findings revealed ML355 could reduce HCC recurrence via the inhibition of ALOX12–12-HETE pathway.

### 12-HETE enhanced migration of HCC cells via EMT and MMPs

**Fig. 4:**
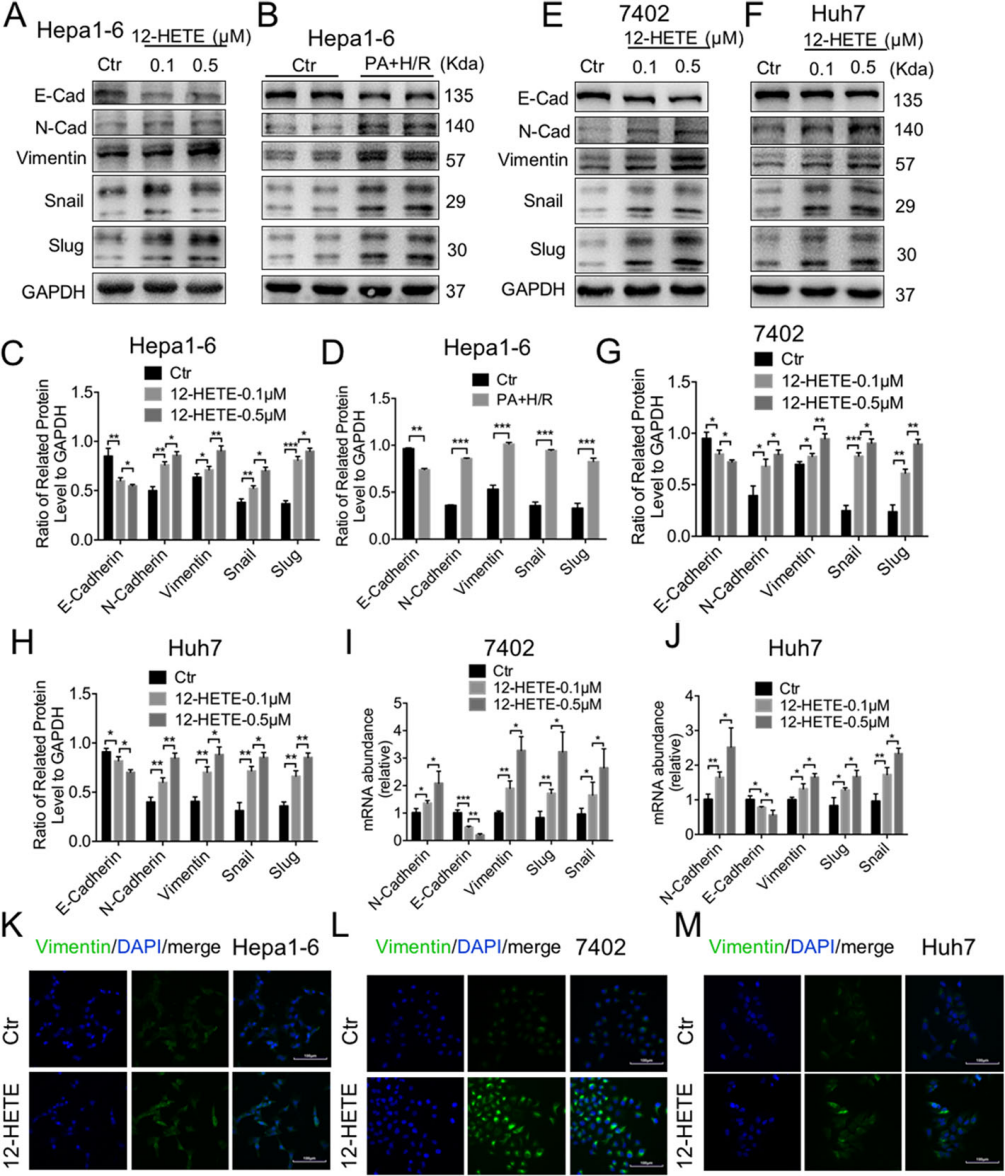
12-HETE enhanced migration of HCC cells via activation of EMT. **a, c** Immunoblot analysis of E-cadherin, N-cadherin, Vimentin, Snail and Slug in Hepa1–6 cells stimulated with 12-HETE. Protein levels were normalized to GAPDH and analyzed. (*n* = 3 per group) **b, d.** Immunoblot analysis of E-cadherin, N-cadherin, Vimentin, Snail and Slug in Hepa1–6 cells stimulated with supernatant of AML12 cell. Protein levels were normalized to GAPDH and analyzed. (n = 3 per group) **e-h.** Immunoblot analysis of E-cadherin, N-cadherin, Vimentin, Snail and Slug in bel-7402 and Huh7 cells stimulated with 12-HETE. Protein levels were normalized to GAPDH and analyzed. (n = 3 per group) **i, j.** qPCR analysis of *E-cadherin, N-cadherin, Vimentin, Snail* and *Slug* in bel-7402 and Huh7 cells stimulated with 12-HETE. (n = 4–5 per group) **k-m.** Representative immunofluorescence staining of Vimentin in bel-7402, Hepa1–6 and Huh7 cells. Scale bars, 100 μm. Data are mean ± SEM, *p < 0.05, **p < 0.01, ***p < 0.001 by unpaired Student’s t- test

EMT is considered to be a key step for tumor invasion and metastasis, and it has been implicated in HCC recurrence. Thus, we next examined whether 12-HETE promoted HCC recurrence through activating EMT signaling pathway. We first stimulated Hepa1–6 cell with 12-HETE (Cayman, USA), and found that N-cadherin, Vimentin, Snail and Slug were upregulated, and E-Cadherin was downregulated (Fig. 4a, c). Also, EMT was induced by 12-HETE in a concentration-dependent manner (Fig. 4a, c). Then, we stimulated Hepa1–6 cell with the supernatant of AML12 cell induced by PA and H/R, and found that EMT was also activated in Hepa1–6 cells (Fig. 4b, d). Next, we used two human HCC cell lines, bel-7402 and Huh7, to verify the role of 12-HETE on EMT. Both protein and mRNA levels of EMT related genes were upregulated by 12-HETE in these cells (Fig. 4e-j), and the immunofluorescence staining of Vimentin was also increased (Fig. 4k-m). These results suggested that 12-HETE could induce EMT and help the migration of circulation tumor cells.

**Fig. 5:**
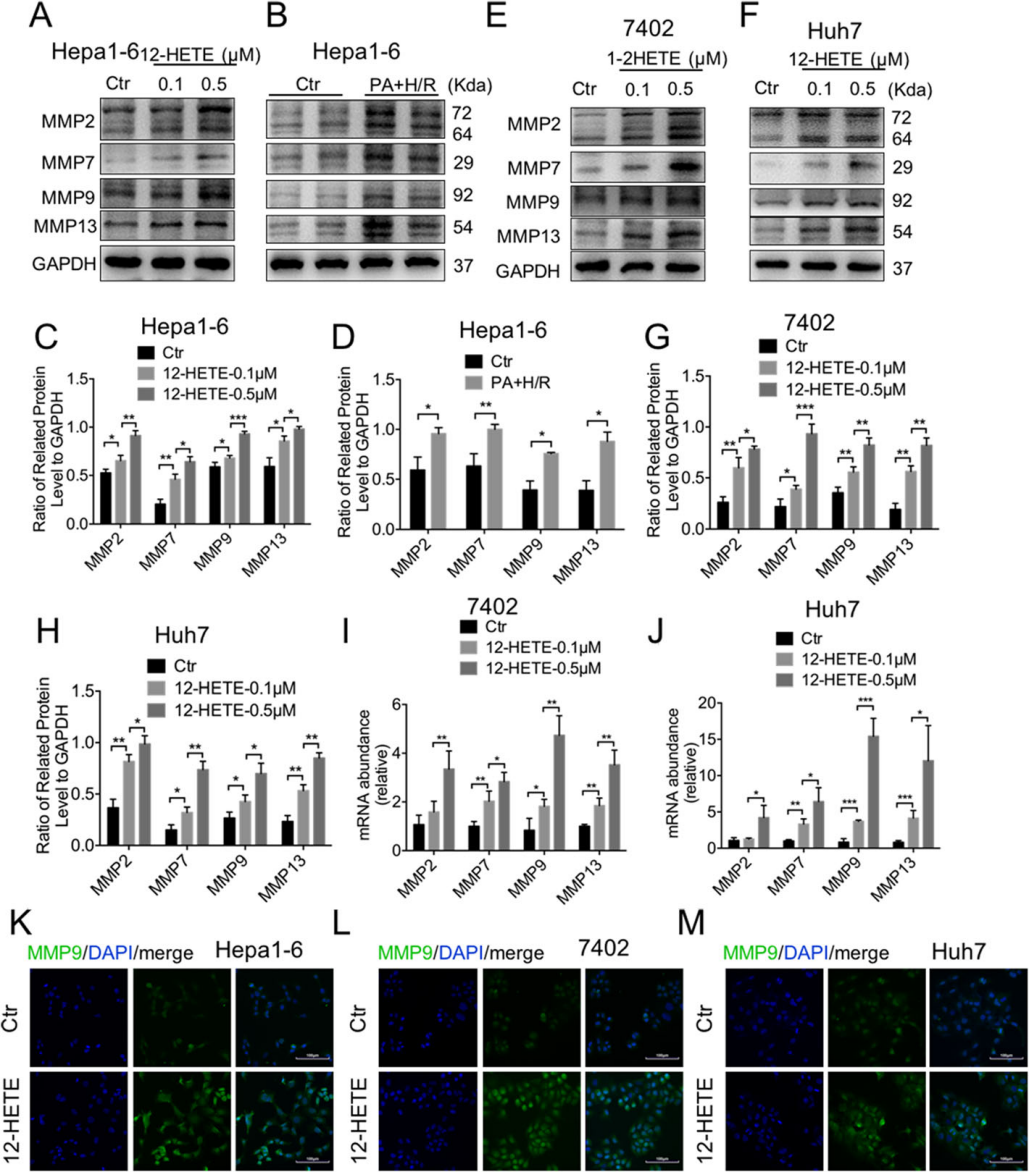
12-HETE enhanced migration of HCC cells via activation of MMPs. **a, c** Immunoblot analysis of MMP2, MMP7, MMP9 and MMP13 in Hepa1–6 cells stimulated with 12-HETE. Protein levels were normalized to GAPDH and analyzed. (n = 3 per group) **b, d** Immunoblot analysis of MMP2, MMP7, MMP9 and MMP13 in Hepa1–6 cells stimulated with supernatant of AML12 cell. Protein levels were normalized to GAPDH and analyzed. (n = 3 per group) **e-h** Immunoblot analysis of MMP2, MMP7, MMP9 and MMP13 in bel-7402 and Huh7 cells stimulated with 12-HETE. Protein levels were normalized to GAPDH and analyzed. (n = 3 per group) **i, j.** qPCR analysis of *MMP2, MMP7, MMP9* and *MMP13* in bel-7402 and Huh7 cells stimulated with 12-HETE. (n = 4–5 per group) **k-m** Representative immunofluorescence staining of MMP9 in bel-7402, Hepa1–6 and Huh7 cells. Scale bars, 100 μm. Data are mean ± SEM, *p < 0.05, **p < 0.01, ***p < 0.001 by unpaired Student’s t- test

Except for EMTs, MMPs are also found to contribute to tumor recurrence, and 12-HETE could induce MMP9 expression. However, whether 12-HETE could upregulate MMPs in HCC cells is unclear. Therefore, we stimulated Hepa1–6 cell with 12-HETE, and found that MMP2, MMP7, MMP9 and MMP13 were all upregulated in a concentration-dependent manner (Fig. 5a, c). The supernatant of AML12 cells also activated MMPs in Hepa1–6 cells induced by PA and H/R (Fig. 5b, d). Moreover, the mRNA and protein levels of MMPs were upregulated in both bel-7402 and Huh7 cells (Fig. 5e-j). We also detected MMP9 by immunofluorescence, and found that MMP9 staining was increased (Fig. 5k-m). Overall, 12-HETE enhanced the invasion and migration of circulation tumor cells by activating EMT and MMPs, which may contribute to HCC recurrence.

### 12-HETE induced EMT and MMPs through the activation of PI3K/AKT/NF-κB pathway

**Fig. 6:**
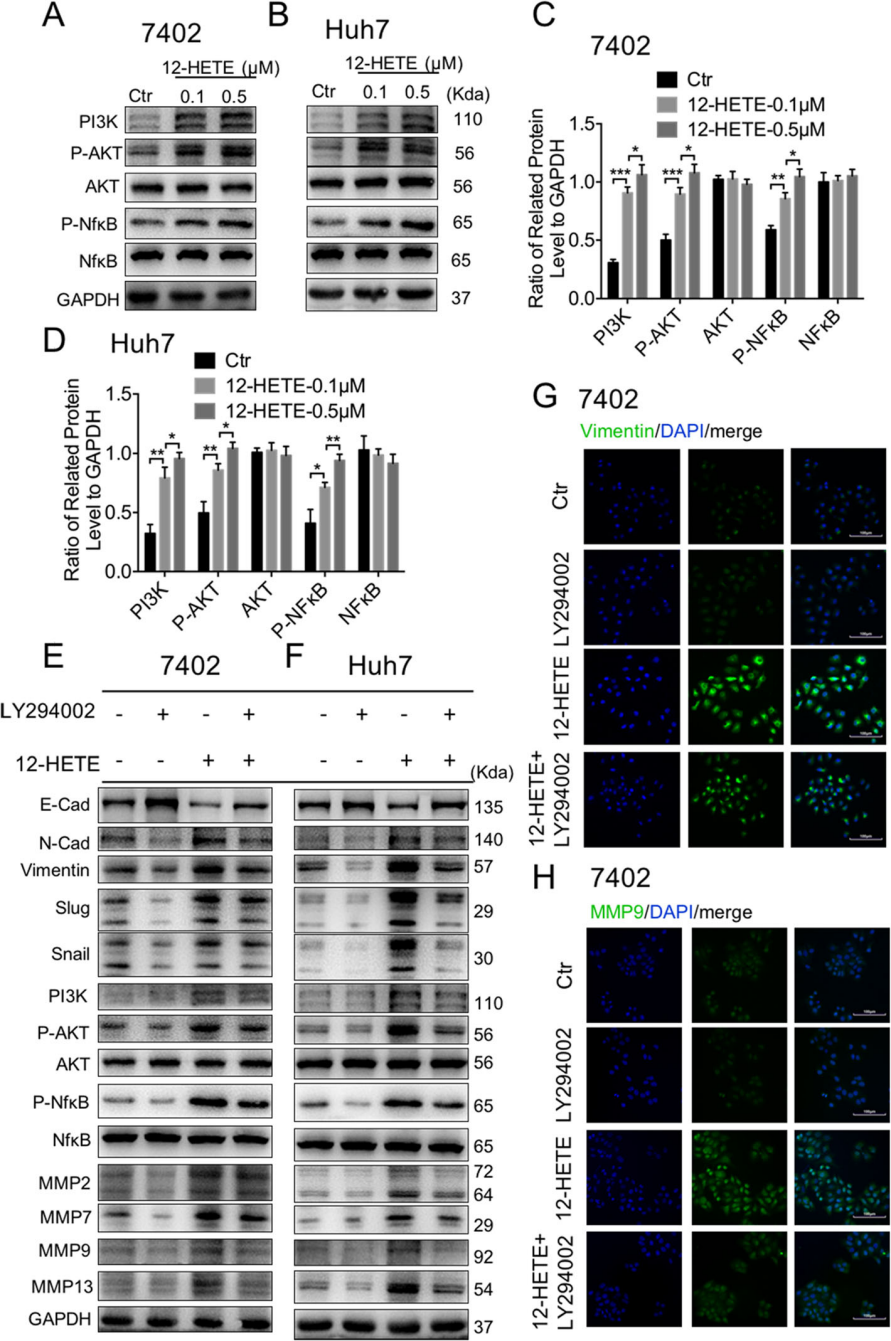
12-HETE induced EMT and MMPs through activation of PI3K/AKT/NF-κB pathway. **a-d** Immunoblot analysis of PI3K, AKT and NFκB in bel-7402 and Huh7 cells stimulated with 12-HETE. Protein levels were normalized to GAPDH and analyzed. (n = 3 per group) **e, f** Immunoblot analysis of PI3K, AKT, NFκB, E-cadherin, N-cadherin, Vimentin, Snail, Slug, MMP2, MMP7, MMP9 and MMP13 in bel-7402 and Huh7 cells stimulated with LY294002 and 12-HETE. **g, h** Representative immunofluorescence staining of Vimentin and MMP9 in bel-7402 cells. Scale bars, 100 μm. Data are mean ± SEM, *p < 0.05, **p < 0.01, ***p < 0.001 by unpaired Student’s t- test

The PI3K/Akt/NF-κB pathway plays a pivotal role in many cellular processes, such as survival, proliferation, cell cycle control, angiogenesis and invasiveness. Therefore, we next investigated if 12-HETE induced EMT and MMPs by activating the PI3K/AKT/NF-κB signaling pathway. In bel-7402 and Huh7 cells, 12-HETE could induce PI3K/AKT/NF-κB pathway in a concentration-dependent manner (Fig. 6a-d). We also treated the cells with LY294002, a PI3K/AKT inhibitor, with or without 12-HETE stimulation. PI3K/AKT/NF-κB signaling pathway was inhibited (Fig. 6e and f, Additional file 2: Fig. S1A and B) by LY294002, as well as EMT and MMPs related markers. Also, Vimentin and MMP9 were significantly suppressed by LY294002 in bel-7402, Huh7 and Hepa1–6 cells, as shown by immunofluorescence staining (Fig. 6g and h, Additional file 3: Fig. S2A-D). In summary, 12-HETE could induce EMT and MMPs through the activation of PI3K/AKT/NF-κB pathway, which enhances the invasion and migration of circulation tumor cells.

### GPR31 mediates the IRI induced HCC recurrence in NAFLD

**Fig. 7:**
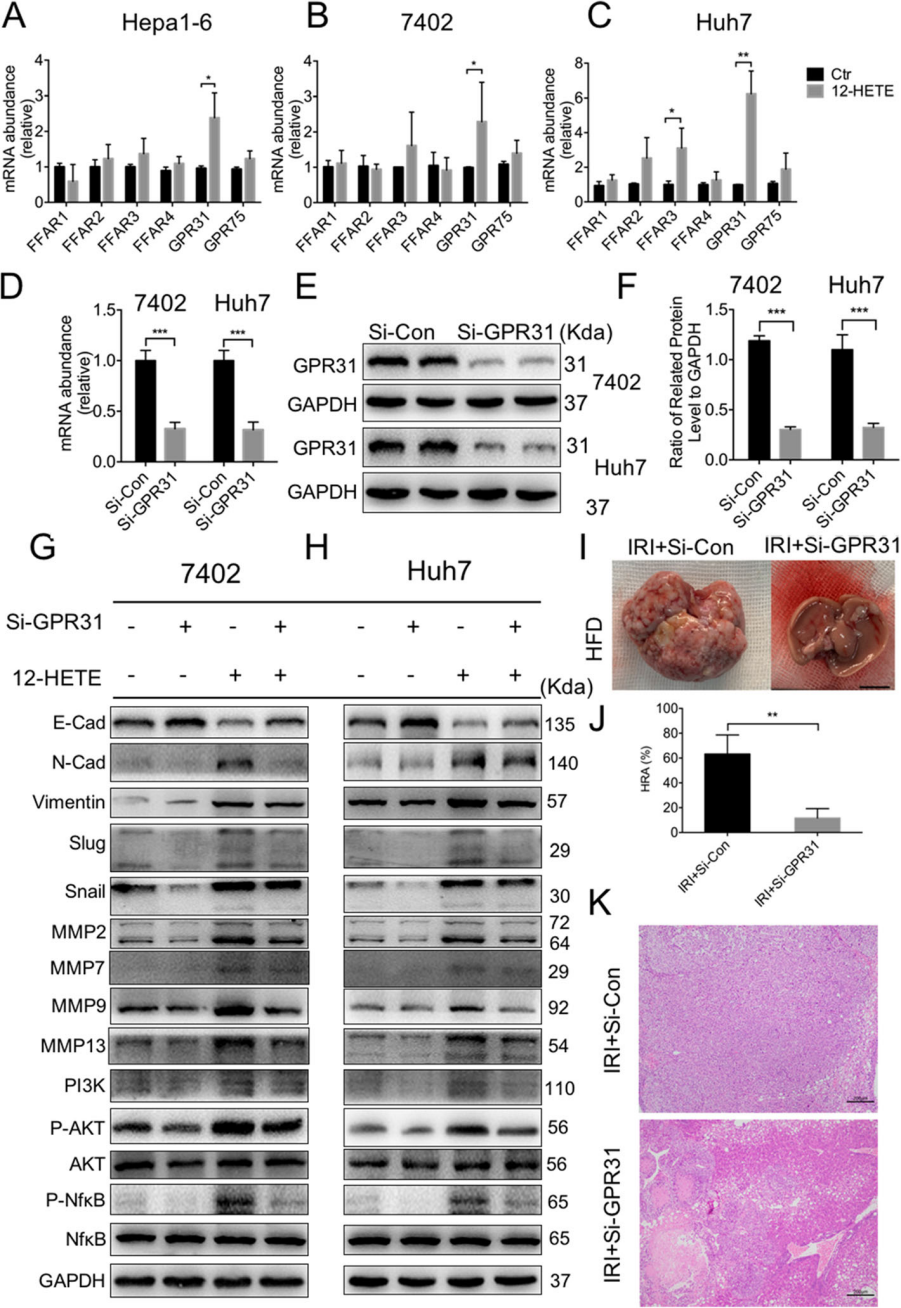
GPR31 mediates the IRI induced HCC recurrence in NAFLD. **a-c** qPCR analysis of *FFAR1, FFAR2, FFAR3, FFAR4, GPR31*and *GPR75* in Hepa1–6, bel-7402 and Huh7 cells stimulated with 12-HETE. (n = 4–5 per group). **d-f** qPCR and immunoblot analysis of GPR31 in bel-7402 and Huh7 cells with Si-GPR31 or Si-Con. Protein levels were normalized to GAPDH and analyzed. (n = 3–4 per group) **g, h.** Immunoblot analysis of PI3K, AKT, NFκB, E-cadherin, N-cadherin, Vimentin, Snail, Slug, MMP2, MMP7, MMP9 and MMP13 in bel-7402 and Huh7 cells with Si-GPR31 and 12-HETE stimulation. **i, j.** Representative images and HRA of HCC recurrence in HFD mice suffered IRI and inoculated with GPR31^−/−^ Hepa1–6 cells. Scale bars, 1 cm. (n = 3–4 per group) **f** Representative H&E staining of liver sections of HCC recurrence in HFD mice suffered IRI and inoculated with GPR31^−/−^ Hepa1–6 cells. Scale bars, 200 μm. Data are mean ± SEM, *p < 0.05, **p < 0.01, ***p < 0.001 by unpaired Student’s t- test

Next, we asked how 12-HETE promotes HCC recurrence in NAFLD. As a lipid metabolite, 12-HETE binds to specific receptor and mediates the downstream signal transduction. As G Protein-Coupled Receptors (GPCR) are known to be responsive to long-chain fatty acids, we examined the changes of GPCR under 12-HETE stimulation. As shown in Fig. 7a-c, GPR31 was upregulated in Hepa1–6, bel-7402 and Huh7 cells, while FFAR3 was only slightly increased in Huh7 cell with 12HETE stimulation. To further verify the role of GPR31, we used siRNA to knockdown GPR31 in both bel-7402 and Huh7 cells (Fig. 7d-f), and found that EMT and MMPs were inhibited when GPR31 was suppressed, indicating that GPR31 may play an important role in 12-HETE induced HCC recurrence (Fig. 7g and h, Additional file 4: Fig. S3A and B). Thus, we knocked out GPR31 in Hepa1–6 cells (Additional file 5: Fig. S4A-C) and examined the HCC recurrence in vivo. As shown in Fig. 7i, GPR31 knockdown significantly reduced the HCC recurrence. The livers exhibited decreased number and volume of nodules, as shown by HE staining (Fig. 7j and k). In conclusion, GPR31 is responsible for 12-HETE-mediated HCC recurrence in NAFLD.

## Discussion

Numerous studies have shown the negative effect of IRI on the recurrence of liver cancer [26–28]. In addition, although not uniformly [29], previous results from both experimental and clinical research have found that fatty livers are prone to cancer growth and recurrence after oncological surgery [30, 31]. Obesity has also been reported to be associated with an earlier and increased risk of HCC recurrence after liver transplantation [32]. What’s more, in the past two decades, urbanization has led to sedentary lifestyle and overnutrition, which promote the increase in obesity and NAFLD. The currently estimation of the NAFLD ratio is 24% worldwide, which may lead to more steatosis donors for liver transplantation [33, 34]. Our study also found more severe HCC recurrence in NAFLD, but the underlying mechanism was unclear, and no effective method has been developed to reduce the recurrence rate. Hypoxia, ATP depletion, oxidative stress and DAMPs-mediated activation of the innate immune response all contributed to liver IRI [35]. The components of arachidonic acid metabolic pathway, ALOX12 and its product 12-HETE, were activated during the early stages of hepatic IRI [36]. Recent studies showed that ALOX12–12-HETE was upregulated in NAFLD [37]. But whether ALOX12–12-HETE was activated during IRI in fatty livers remains unknown. Our study found that the activation of ALOX12–12-HETE was further enhanced in NAFLD during IRI than the normal livers, and the inhibition of ALOX12 by ML355 decreased 12HETE levels. The role of ALOX12 has been characterized in cell migration, proliferation, and platelet aggregation in the context of neoplasia and atherosclerosis. Elevated 12-HETE was strongly associated with the presence of HCC [38], and it could activate the 12-HETER–RHO–ROCK–MYPT signaling cascade to induce MLC2 function and enhance metastasis [39]. Therefore, we hypothesized that IRI induced ALOX12–12-HETE activation, which promoted HCC recurrence in NAFLD. Our hypothesis was supported by the reduced HCC recurrence in NALFD when ALOX12 was inhibited by ML355.

EMT and MMPs are two mechanisms for cancer invasion and metastasis. MMPs degrade proteins in the extracellular matrix (ECM) and dissolve the connective tissue through proteolysis, thus removing the barrier for tumor cells to migrate through local tissues [40]. High MMPs expression in tumors have been associated with poor prognosis in patients with various types of cancer [41, 42], including HCC [43]. Also, MMP-9 and MMP-2 played an important role in the prediction of tumor recurrence and survival in HCC patients after surgical resection [44]. EMT is a reversible cellular program that transiently places epithelial cells into a quasi-mesenchymal cell states [45, 46]. Moreover, the malignant progression of many types of carcinoma depends on EMT activation in neoplastic cells [47]. However, the role of EMT and MMPs in HCC recurrence in NAFLD has not been fully studied. In our study, we found that EMT and MMPS were activated under 12-HETE stimulation in a concentration-dependent manner. PI3K-AKT signaling pathway is an independent factor that predicted poor survival and high recurrence rate in HCC patients [48]. We also found that PI3k-AKT-NFκB pathway was activated under the stimulation of 12-HETE, and inhibiting PI3k pathway by LY294002 could suppress the activation of EMT and MMPs. Thereby, the activation of EMT and MMPs by 12-HETE might play an important role in HCC recurrence in NAFLD.

**Fig. 8:**
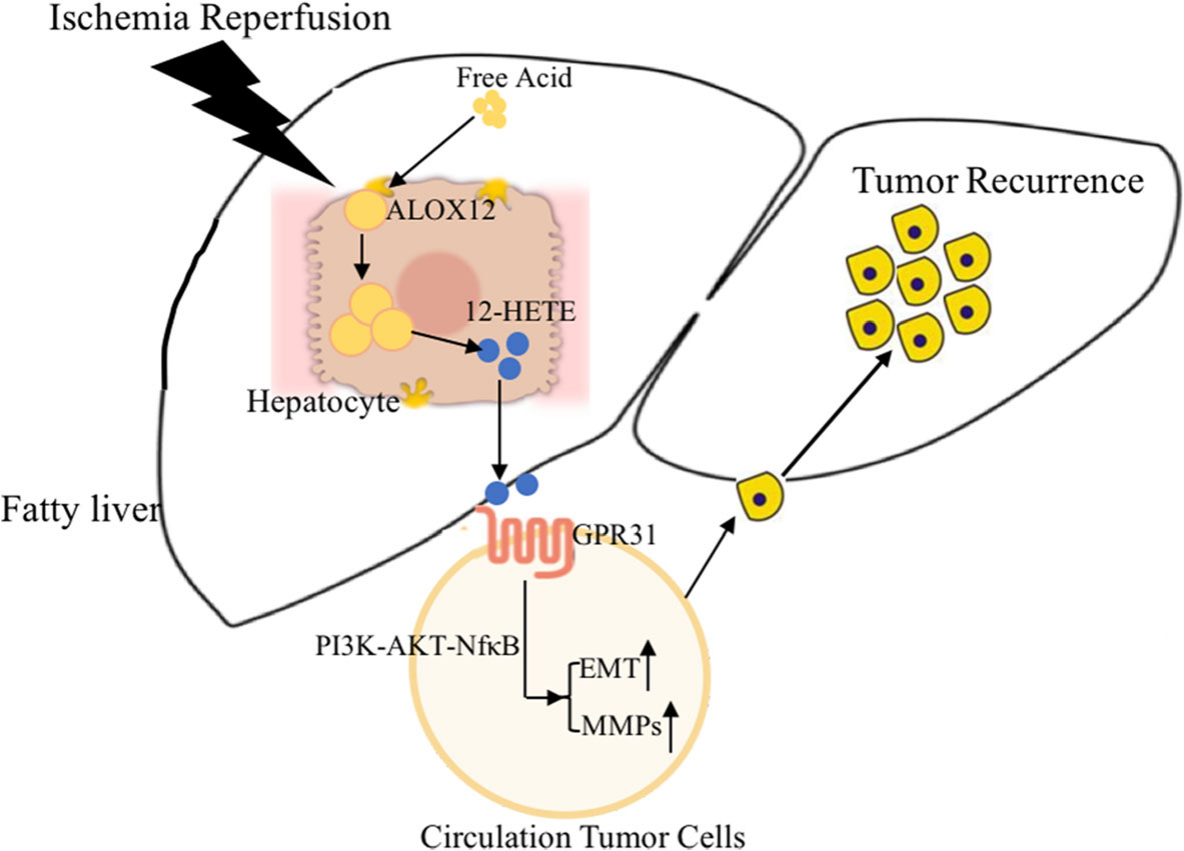
Ischemia reperfusion injury promotes recurrence of hepatocellular carcinoma in NAFLD via ALOX12–12HETE-GPR31 signaling axis. ALOX12–12-HETE pathway was activated during liver IRI and enhanced in NAFLD, which induced more severe HCC recurrence in fatty liver than normal. 12-HETE induced GPR31 dependent activation of EMT and MMPs via PI3K/AKT/NF-κB to enhance invasion ability of circulation tumor cells, which promoted more severe HCC recurrence in NAFLD

Numerous evidence have indicated that GPCR is important for 12-HETE mediated signal transduction [49]. Thus, GPCR might be involved in the recurrence of HCC. We found that GPR31 was the most significantly upregulated receptor among six types of GPCRs. A recent study also found that GPR31 could directly recognize 12-HETE and mediate its function during hepatic IRI [14]. Moreover, as a high-affinity 12-HETE receptor, GPR31 was significantly up-regulated in prostate cancer and played a critical role in prostate cancer progression [50]. Knockdown of GPR31 with siRNA could inhibit the activation of PI3k-AKT-NFκB signaling pathway, as well as EMT and MMPs. Also, knockout of GPR31 in Hepa1–6 cells significantly reduced the HCC recurrence in NAFLD. Therefore, ALOX12–12-HETE-GPR31 played an important role in IRI induced HCC recurrence in NAFLD (Fig. 8).

Recurrence of HCC in NAFLD was a complex process. Both pre-metastasis microenvironment and invasion of circulation tumor cells played an important role in tumor recurrence [51]. In this study, we focused on 12-HETE released into the blood stream and the effect of 12-HETE on circulation tumor cells. However, we didn’t investigate the inflammatory milieu in situ as well as in plasmatic and how this may impact HCC recurrence in this study. The inflammatory microenvironment changed during the ischemia-reperfusion, and this may provide pre-metastasis microenvironment in HCC recurrence. So, further study should be designed to explore how inflammatory milieu may impact HCC recurrence in NAFLD.

## Conclusion

Our study discovers a new mechanism of how IRI enhances HCC recurrence in NAFLD. We demonstrated the important role of ALOX12–12-HETE-GPR31 in HCC recurrence, which may provide a potential therapeutic target to reduce the tumor recurrence in NAFLD after liver transplantation.

## Supplementary information

**Additional file 1: Table S1.** Oligonucleotide primers for quantitative qRT-PCR analysis.

**Additional file 2: Figure S1.** Proteins levels of EMT and MMPs were normalized to GAPDH and analyzed. **A, B** PI3K, AKT, NFκB, E-cadherin, N-cadherin, Vimentin, Snail, Slug, MMP2, MMP7, MMP9 and MMP13 were normalized to GAPDH and analyzed in bel-7402 and Huh7 cells stimulated with LY294002 and 12-HETE. (*n* = 3 per group) Data are mean ± SEM, *p < 0.05, **p < 0.01, ***p < 0.001 by unpaired Student’s t- test.

**Additional file 3: Figure S2.** Immunofluorescence staining of Vimentin and MMP9 in Huh7 and Hepa1–6 cells. **A, B** Immunofluorescence staining of Vimentin in Huh7 and Hepa1–6 cells. **C, D** Immunofluorescence staining of MMP9 in Huh7 and Hepa1–6 cells. Scale bars, 100 μm.

**Additional file 4: Figure S3.** Proteins levels of EMT and MMPs were normalized to GAPDH and analyzed. **A, B** PI3K, AKT, NFκB, E-cadherin, N-cadherin, Vimentin, Snail, Slug, MMP2, MMP7, MMP9 and MMP13 were normalized to GAPDH and analyzed in bel-7402 and Huh7 cells with Si-GPR31 and 12-HETE stimulation. (n = 3 per group) Data are mean ± SEM, *p < 0.05, **p < 0.01, ***p < 0.001 by unpaired Student’s t- test.

**Additional file 5: Figure S4.** Knockdown of GPR31 in Hepa1–6 cells. **A-C** qPCR and immunoblot analysis of GPR31 in bel-7402 and Huh7 cells with Si-GPR31 or Si-Con. Protein levels were normalized to GAPDH and analyzed. (n = 3–4 per group). Data are mean ± SEM, ***p < 0.001 by unpaired Student’s t- test.

## Data Availability

All data generated or analyzed during this study are included in this published article and its additional files.

## Abbreviations

12-HETEs: 12-hydroxyeicosatetraenoic acids
ALOX12: 12-Lipoxygenase
CD: control diet
EMT: epithelial-mesenchymal transition
FFAR: free fatty acid receptor
GPR31: G-protein-coupled receptor 31
GPR75: G-protein-coupled receptor 75
H/R: hypoxia/re-oxygenation
HCC: hepatocellular carcinoma
HFD: high fat diet
IRI: ischemia reperfusion injury
MMPs: matrix metalloprotein
NAFLD: nonalcoholic fatty liver disease
NF-κB: nuclear factor κ B
PI3K: phosphoinositide 3-kinase
