# Supplementary material for: Ischemia reperfusion injury promotes recurrence of hepatocellular carcinoma in fatty liver via ALOX12-12HETE-GPR31 signaling axis
Source: J Exp Clin Cancer Res. 2019 Dec 12;38:489. doi: 10.1186/s13046-019-1480-9 (PMC6909624; doi:10.1186/s13046-019-1480-9)
Supplement: Supplementary file 1 — Additional file 1: Table S1. Oligonucleotide primers for quantitative qRT-PCR analysis. [file 13046_2019_1480_MOESM1_ESM.doc]

Table S1 Oligonucleotide primers for quantitative qRT-PCR analysis

mouse

| Gene | Forward primer (5’-3’) | Reverse primer (5’-3’) |
| --- | --- | --- |
| *β-Actin* | AGTGTGACGTTGACATCCGTA | GCCAGAGCAGTAATCTCCTTCT |
| *ALOX12* | CGCTACCTGATGTGCAGAAG | TCCGGGCTGTAATCTCTTTC |
| *Bv8* | CACACGCCCACCAAGTAGG | TAGCGGGCGAGGGCAGCAATGAA |
| *S100a8* | TGCGATGGTGATAAAGTGG | GGCCAGAAGCTCTGCTACTC |
| *S100a9* | AGACCATCATCAACACCTTCCA | GTCACCCTCGTGCATCTTCTC |
| *VEGF* | GGAGACTCTTCGAGGAGCACTT | GGCGATTTAGCAGCAGATATAAGAA |
| *FFAR1* | TCTGCCTGGGGCCCTATAAT | GTCCAGGACCTGTTCCCAAG |
| *FFAR2* | GGCTCAGAAGCAAGGTGACT | TGTGGCTTAGAGCTTTCCCG |
| *FFAR3* | CCTGGTGTGGATACTGAGCC | ACCAGGGGGTCGATACAAGA |
| *FFAR4* | CCAGATCCGAGTGTCCCAAC | TTCGTTCCTGAACAGCGACA |
| *GPR31* | AACTTGCTGTCTCTGAGGGC | ATGAGGCCACTGTTGCAGAA |
| *GPR75* | CACTAAGGCCAGACATGGCA | GCTCCCAGAGACTGAACCAC |

human

| Gene | Forward primer (5’-3’) | Reverse primer (5’-3’) |
| --- | --- | --- |
| *18S* | AACCCGTTGAACCCCATT | CCATCCAATCGGTAGTAGCG |
| *N-Cadherin* | CGAATGGATGAAAGACCCATCC | GGAGCCACTGCCTTCATAGTCAA |
| *E- Cadherin* | TGTAACTTGCAATGGGCAGC | CAAGCTCTCCTGCCATCTCC |
| *Vimentin* | ACGTCTTGACCTTGAACGCA | TCTTGGCAGCCACACTTTCA |
| *Slug* | GAACTGGACACACATACAGTGAT | GGTAGTCCACACAGTGATGG |
| *Snail* | CAGGACTCTAATCCAGAGTTTACCT | ACAGAGTCCCAGATGAGCATTG |
| *MMP2* | CAAGGACCGGTTTATTTGGC | ATTCCCTGCGAAGAACACAGC |
| *MMP7* | ATGAACGCTGGACGGATGGT | TGGAGTGGAGGAACAGTGCT |
| *MMP9* | TTGACAGCGACAAGAAGTGG | GCCATTCACGTC GTCCTTAT |
| *MMP13* | CTGGCCTGCTGGCTCATGCTT | CCTCAGAAAGAGCAGCATCGATATG |
| *FFAR1* | GCCATCACAGCCTTCTGCTA | AGCGGATTAAGCACCACACT |
| *FFAR2* | GGGACAAATGAGGACAGGGG | ATCTGGATGGGAGCCTTCCT |
| *FFAR3* | ACAGTGAGTGACGTCATGGG | CCAGTGATTGCCGGAGAATG |
| *FFAR4* | CCAAAATTTTACAGATCACAAAGGC | CACCACCCAGAAGAAGAGGG |
| *GPR31* | CGTACGCTGTCTACCTGCTC | TTGACCTTAAGCCGAGGGTG |
| *GPR75* | CGATGGCGATGATGCCTCTA | CATCCTGAAGGTGGCCTGTT |
