# Supplementary figures and images for: Ischemia reperfusion injury promotes recurrence of hepatocellular carcinoma in fatty liver via ALOX12-12HETE-GPR31 signaling axis
Source: J Exp Clin Cancer Res. 2019 Dec 12;38:489. doi: 10.1186/s13046-019-1480-9 (PMC6909624; doi:10.1186/s13046-019-1480-9)

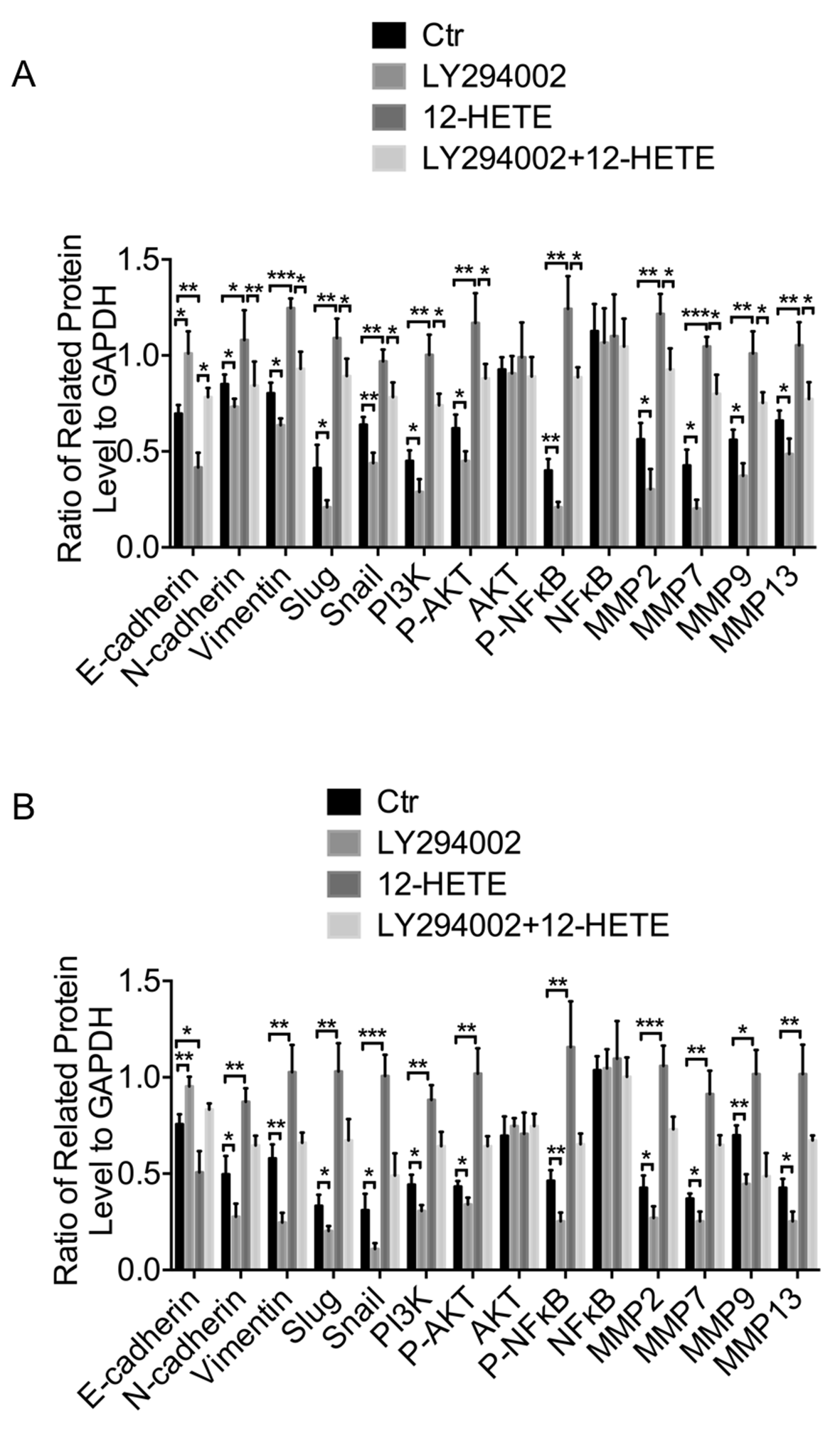

Supplement: Supplementary file 2 — Additional file 2: Figure S1. Proteins levels of EMT and MMPs were normalized to GAPDH and analyzed. A, B PI3K, AKT, NFκB, E-cadherin, N-cadherin, Vimentin, Snail, Slug, MMP2, MMP7, MMP9 and MMP13 were normalized to GAPDH and analyzed in bel-7402 and Huh7 cells stimulated with LY294002 and 12-HETE. (n = 3 per group) Data are mean ± SEM, *p < 0.05, **p < 0.01, ***p < 0.001 by unpaired Student’s t- test. [file 13046_2019_1480_MOESM2_ESM.tif]

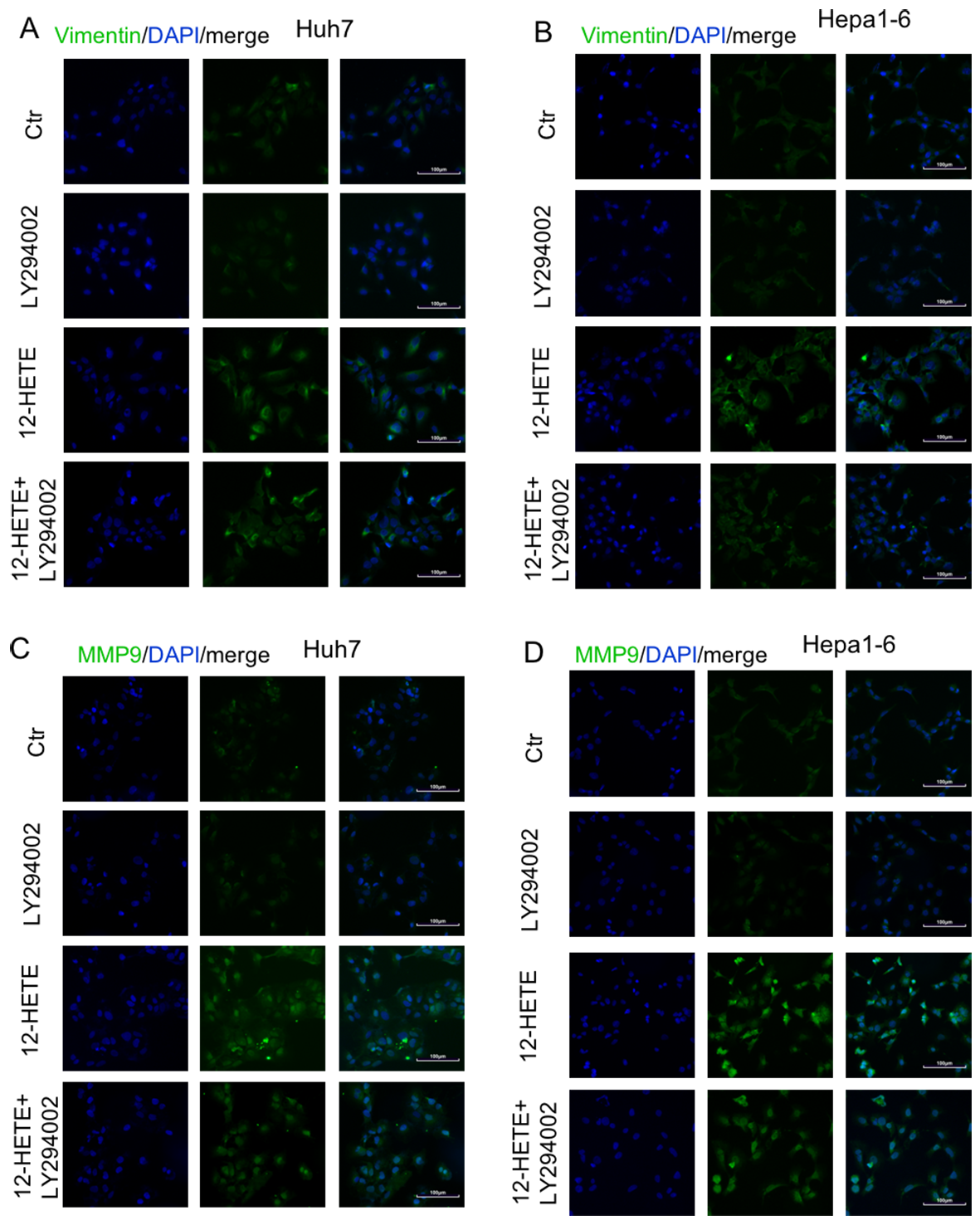

Supplement: Supplementary file 3 — Additional file 3: Figure S2. Immunofluorescence staining of Vimentin and MMP9 in Huh7 and Hepa1–6 cells. A, B Immunofluorescence staining of Vimentin in Huh7 and Hepa1–6 cells. C, D Immunofluorescence staining of MMP9 in Huh7 and Hepa1–6 cells. Scale bars, 100 μm. [file 13046_2019_1480_MOESM3_ESM.tif]

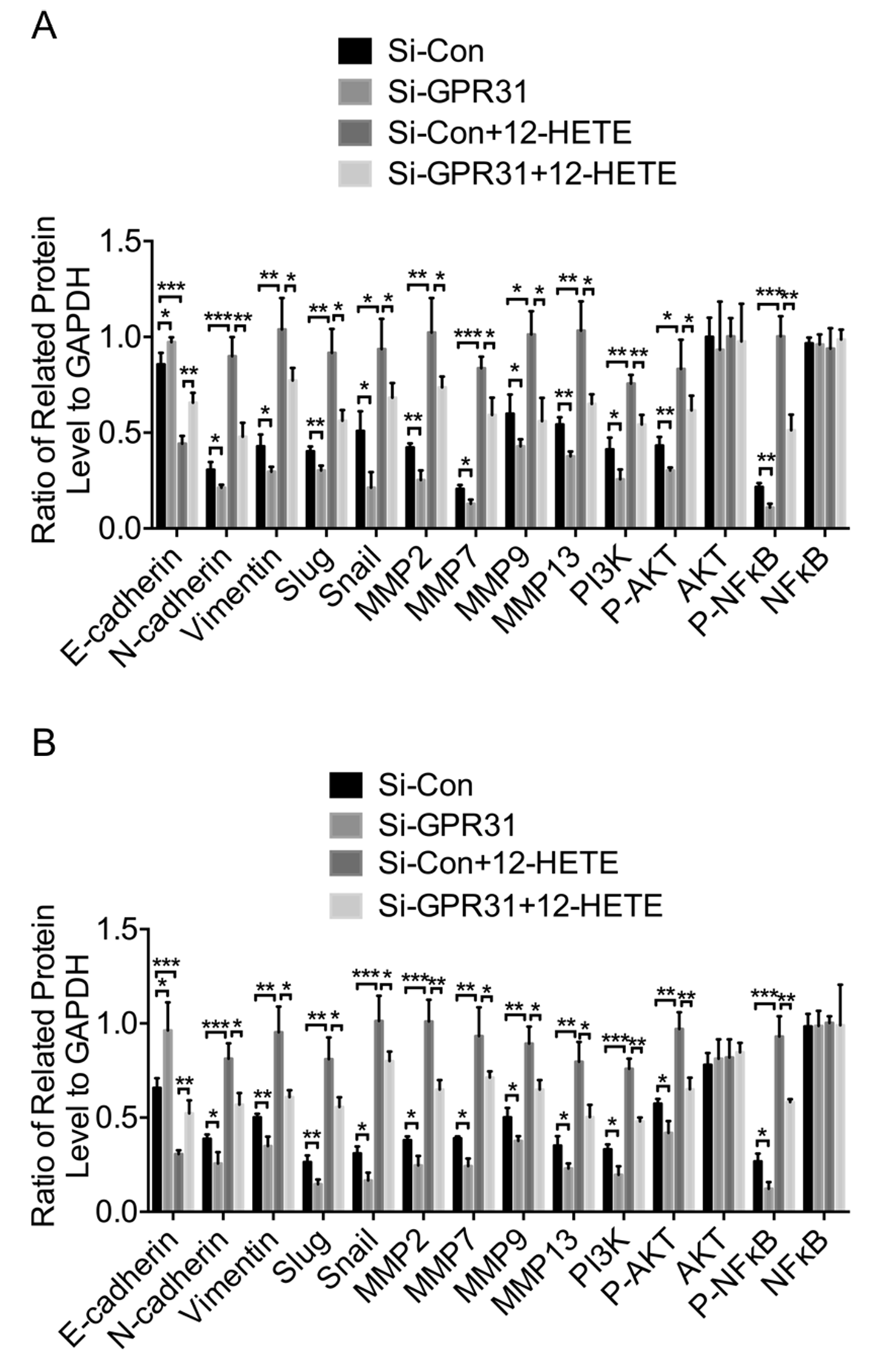

Supplement: Supplementary file 4 — Additional file 4: Figure S3. Proteins levels of EMT and MMPs were normalized to GAPDH and analyzed. A, B PI3K, AKT, NFκB, E-cadherin, N-cadherin, Vimentin, Snail, Slug, MMP2, MMP7, MMP9 and MMP13 were normalized to GAPDH and analyzed in bel-7402 and Huh7 cells with Si-GPR31 and 12-HETE stimulation. (n = 3 per group) Data are mean ± SEM, *p < 0.05, **p < 0.01, ***p < 0.001 by unpaired Student’s t- test. [file 13046_2019_1480_MOESM4_ESM.tif]

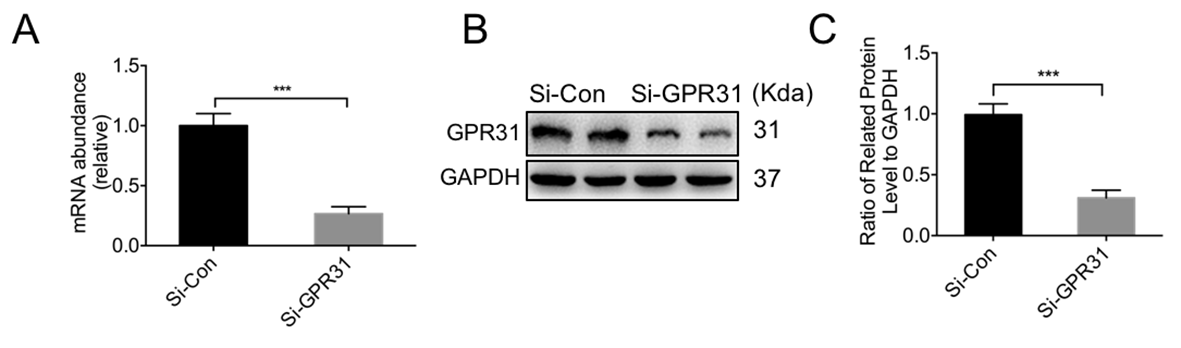

Supplement: Supplementary file 5 — Additional file 5: Figure S4. Knockdown of GPR31 in Hepa1–6 cells. A-C qPCR and immunoblot analysis of GPR31 in bel-7402 and Huh7 cells with Si-GPR31 or Si-Con. Protein levels were normalized to GAPDH and analyzed. (n = 3–4 per group). Data are mean ± SEM, ***p < 0.001 by unpaired Student’s t- test. [file 13046_2019_1480_MOESM5_ESM.tif]
